# Incidence and Metastasis of Cutaneous Malignant Melanoma with Respect to ABO Blood Groups: A Case-Controlled Study in Northeast of China

**DOI:** 10.1371/journal.pone.0088096

**Published:** 2014-02-07

**Authors:** Liang Chang, Junrui Pei, Chenlong Li, Ping Zhang, Dan Zhou, Wenzhong Du, Xing Liu, Chuanlu Jiang

**Affiliations:** 1 Department of Neurosurgery, The Tumor Hospital, Harbin Medical University, Harbin, People’s Republic of China; 2 Chinese Center for Endemic Disease Control, Harbin Medical University, Harbin, People’s Republic of China; 3 The Key Laboratory for Etiological Epidemiology of the Ministry of Health, Harbin Medical University, Harbin, People’s Republic of China; 4 Department of Neurosurgery, The Second Affiliated Hospital, Harbin Medical University, Harbin, People’s Republic of China; University of Tennessee, United States of America

## Abstract

**Background:**

ABO blood groups have been suggested to contribute to the development of certain tumors; however, the associations between ABO blood groups and the incidence and metastases of cutaneous malignant melanomas have not been fully evaluated in Chinese populations. Thus, we investigated these associations with a case-controlled study in northeast of China.

**Methods:**

A total of 482 patients with cutaneous malignant melanoma and 3,068 healthy- controls were enrolled for the study between 2001 and 2012 at The Tumor Hospital of Harbin Medical University. A multivariate logistic model was used to evaluate adjusted odds ratios (ORs) and 95% confidence intervals (CI) for the incidence and metastases of cutaneous malignant melanoma.

**Results:**

Blood type A individuals had higher tumor incidence and metastasis compared to those with blood type O (OR = 1.575; 95% CI = 1.208–2.053, p = 0.001; OR = 2.004; 95% CI = 1.032–3.889, p = 0.040), after adjusting for age, gender, smoking status and alcohol consumption.

**Conclusions:**

Blood type A was associated with higher incidence and metastasis of cutaneous malignant melanoma but future studies are needed to examine the mechanisms linking cutaneous malignant melanoma to ABO blood types.

## Introduction

Derived from abnormal melanocytes, malignant melanoma is the most dangerous primary malignant skin tumor, responsible for more than 75% of skin cancer deaths [1]. Each year, nearly 20,000 cases of cutaneous malignant melanoma are diagnosed in China. Because this type of cancer has high invasiveness, patient mortality is high and five-year survival rates of patients with metastatic disease is less than 20% [1], [2]. Thus, understanding the etiology of this type of cancer and identifying high-risk individuals are essential strategies for preventing and treating this tumor type. The development of melanoma appears to be related to multiple risk factors such as skin and hair color as well as any family history of melanoma [3], [4]. However, the most significant factors associated with the development of this type of tumor remain to be identified.

ABO blood groups were established by Landsteiner in 1900. According to the presence of A, B, or O blood group antigens erythrocyte surfaces, individual blood types of A, B, AB, or O exist. Also, ABO blood group antigens are expressed in various human issues. Recently, increasing attention has been directed at a possible association between ABO blood groups and the risk of certain malignancies, such as hepatocellular carcinoma [5], and gastric and pancreatic cancer [6], [7]. Several plausible mechanisms, including inflammation, immune-surveillance for malignant cells and intercellular adhesion have been proposed to explain such associations [8]–[10]. Thus, to further understand the potential association between ABO blood groups and the development of cutaneous malignant melanoma in a Chinese population, we conducted a case-controlled study.

## Materials and Methods

### Ethics Statement

This is a retrospective study and written consent was given by the patients for their information to be stored in the hospital database and used for research. Approval was obtained from the Ethics Committee of The Tumor Hospital, Harbin Medical University, Harbin, People’s Republic of China.

### Study Population

A total of 482 patients with cutaneous malignant melanoma were enrolled for the study between 2001 and 2012 at The Tumor Hospital of Harbin Medical University, Harbin, People’s Republic of China. We excluded participants with other tumors. The inclusion criteria for cases were as follows: definitive histopathological confirmation of cutaneous malignant melanoma; definitive anatomic site of cutaneous malignant melanoma; data regarding blood type; sex, age, alcohol consumption and smoking status as obtained through medical records, and a residence within northeast China of more than ten years. In addition, 3,068 healthy-controls, admitted to the hospital for a checkup, were randomly selected during the same period at the same hospital. The inclusion criteria for controls were the same as those for cases, except for the cancer diagnosis.

### Data Collection

Detailed information for all patients was collected from individual medical records. Melanoma stages were determined according to the American Joint Committee on Cancer staging system. Smokers and non-smokers were segregated based on whether the person had smoked more than 100 cigarettes during his lifetime [11]. Alcohol consumers were segregated by drinking habits. We classified alcohol consumption as “No/moderate” (0–25 g/day) and “Yes” (>25 g/day) [11]. Anatomic site of cutaneous malignant melanoma was categorized into “Head & neck”, “Trunk” or “Limbs” [12]–[14].

### Statistical Analysis

The SPSS 14.0 statistical software program was used for statistical analysis. Age, gender, smoking status and alcohol consumption between the cases and healthy-controls, as well as metastatic and non-metastatic cases were assessed with Pearson’s chi-square test. We also used Pearson’s chi-square test to compare proportions regarding anatomic site of tumors among cases with various blood groups. To evaluate associations between ABO blood groups and the incidence for cutaneous malignant melanoma, as well as ABO blood types and metastases in cutaneous malignant melanoma patients, crude and adjusted odds ratios (OR) and 95% confidence intervals (CI) for each variable were calculated by using unconditional logistic regression. P<0.05 was considered statistically significant.

## Results

### Characteristics of Cases and Healthy-controls Controls

The characteristics of cases and healthy-controls are shown in Table 1. No statistically significant differences were found when comparing these variables between patients and controls, suggesting that frequency matching was adequate.

**Table 1:** Characteristics of malignant melanoma patients and healthy controls.

	Cases	Controls	
Variable	N = 482 (%)	N = 3068 (%)	P value^1^
**Age**			0.837
<65	310 (64.3)	1988 (64.8)	
≥65	172 (35.7)	1080 (35.2)	
**Gender**			0.572
Male	307 (63.7)	1913 (62.4)	
Female	175 (36.3)	1155 (37.6)	
**Smoking** **(current or former)**			0.073
No	330 (68.5)	1972 (64.3)	
Yes	152 (31.5)	1096 (35.7)	
**Alcohol consumption**			0.063
No/moderate	303 (62.9)	1794 (58.4)	
Yes	179 (37.1)	1279 (41.6)	

1 Pearson’s chi-square test.

### ABO Blood Groups and Incidence of Cutaneous Malignant Melanoma

The association between ABO blood types and the incidence of cutaneous malignant melanoma was estimated using unconditional logistic regression analysis (Table 2). Blood type A seemed to be correlated to the incidence of cutaneous malignant melanoma. Adjusting for age, gender, smoking status and alcohol consumption using multivariate logistic regression blood type A was associated with the incidence of cutaneous malignant melanoma and no other blood type was associated in this way.

**Table 2:** The association between cutaneous malignant melanoma risk and ABO blood types: Univariate and multivariate logistic regression analyses.

	Cases	Controls				
ABO blood type	N = 482 (%)	N = 3068 (%)	Univariate OR (95% CI)	P value	Multivariate OR (95% CI)^1^	P value^1^
O	105 (21.7)	752 (24.5)	1 (reference)		1 (reference)	
A	178 (36.9)	859 (28.0)	1.494 (1.152−1.938)	0.002	1.575 (1.208−2.053)	0.001
B	163 (33.7)	1269 (41.4)	0.92 (0.708−1.195)	0.531	0.980 (0.750−1.281)	0.883
AB	36 (7.5)	189 (6.2)	1.364 (0.905−2.057)	0.138	1.419 (0.930−2.167)	0.104

1 Adjusted by age, gender, smoking status and alcohol consumption. CI, confidence interval; OR, odds ratio.

Anatomic site of cutaneous malignant melanoma in the present study grouped by blood type are shown in Table 3. No significant differences in the anatomic site of tumors among patients with various blood groups were evident (P = 0.953).

**Table 3:** Anatomic site of cutaneous malignant melanoma according to ABO blood type.

	Blood type			
Anatomic site	O(%)	A(%)	B(%)	AB(%)
Head & neck	29(27.6)	52(29.2)	49(30.1)	8(22.2)
Trunk	45(42.9)	70(39.3)	61(37.4)	16(44.4)
Limbs	31(29.5)	56(31.5)	53(32.5)	12(33.3)

### ABO Blood Groups and Metastasis in Cutaneous Malignant Melanoma

Metastatic and non-metastatic patient characteristics are shown in Table 4 and age, gender, smoking status and alcohol consumption did not create statistical differences between the two groups. We then investigated the association between ABO blood types and metastases in cutaneous malignant melanoma (non-metastatic patients were controls) using unconditional logistic regression analysis (Table 5). Subjects with blood type A were more likely to have metastases for cutaneous malignant melanoma and this was statistically significant different. In contrast, no association was observed between other blood types (B and AB) and metastases in cutaneous malignant melanoma (ORs are depicted in Table 5). Adjusting for age, gender, smoking status and alcohol consumption using multivariate logistic regression, we observed that blood type A was associated with metastases for cutaneous malignant melanoma and no other blood types (B and AB) were associated in this way.

**Table 4:** Characteristics s in patients with metastasis and without metastasis.

	M0	M1	
Variable	N = 378 (%)	N = 104 (%)	P value^1^
**Age**			0.111
<65	250 (66.1)	60 (57.7)	
≥65	128 (33.9)	44 (42.3)	
**Gender**			0.158
Male	237 (62.7)	73 (70.2)	
Female	141 (37.3)	31 (29.8)	
**Smoking** **(current or former)**			0.086
No	266 (70.4)	64 (61.5)	
Yes	112 (29.6)	40 (38.5)	
**Alcohol consumption**			0.548
No/moderate	235 (62.2)	68 (65.4)	
Yes	143(37.8)	36 (34.6)	

1 Pearson’s chi-square test: M0, no metastasis; M1, metastasis.

**Table 5:** The association between ABO blood types and metastasis in patients with cutaneous malignant melanoma: Univariate and multivariate logistic regression analyses.

	M0	M1				
ABO blood type	N = 378 (%)	N = 104 (%)	Univariate OR (95% CI)	P value	Multivariate OR (95% CI)^1^	P value^1^
O	87 (23.0)	18 (17.3)	1 (reference)		1 (reference)	
A	128 (33.9)	50 (48.1)	1.888 (1.032−3.453)	0.039	2.004 (1.032−3.889)	0.040
B	133 (35.2)	30 (28.8)	1.090 (0.573−2.075)	0.793	1.070 (0.514−2.226)	0.857
AB	29 (7.7)	7 (6.7)	1.167 (0.443−3.074)	0.755	1.019 (0.340−3.049)	0.973

1 Adjusted by age, gender, smoking status and alcohol consumption. CI, confidence interval; OR, odds ratio; M0, no metastasis; M1, metastasis.

## Discussion

Previous studies suggest a relationship between ABO blood types and the risk of various cancers. For instance, it is widely accepted that there is a relationship between blood group A and gastric cancer [15], and a positive association has been reported between blood type A and the risk of pancreatic cancer [16]. Blood type B has been said to be related to a higher incidence of ovarian cancer [17]. Here, we report that blood group A was associated with a statistically significant increased incidence of cutaneous malignant melanoma, compared with those with blood group O. Blood type A was also associated with increased metastasis in cutaneous malignant melanoma patients. To our knowledge, this is the first case-controlled study to indicate a correlation between ABO blood types and the development of cutaneous malignant melanoma in a Chinese population.

To date, only two studies [18], [19] document the relationship between the risk of cutaneous malignant melanoma and ABO blood groups, but data from each are inconsistent. In a prospective cohort study of 95,470 US participants derived from the Nurses’ Health Study and the Health Professionals Follow-up Study [18], 685 participants developed melanoma during the study follow-up period (27.1 years for females; 16.9 years for males). There was no statistically significant decreased risk of developing melanoma across A, B, and AB blood types, compared to participants with blood group O. Accordingly, Vincenzo and colleagues [19], reported (445 patients with a histological diagnosis of malignant melanoma and 38,321 controls) a positive association between blood type O and the risk of malignant melanoma. Our data conflict with prior studies and possible reasons for this discrepancy may include differences in participants. Our participants were Chinese, living in northeast China. Participants of two prior studies were Caucasian, living in United States or Italy. Racial differences may exist and dwelling latitudes may be associated with ultraviolet radiation exposure. Also, some studies did not adjust for age or other possible confounders. Our demographic factors for cases and controls were comparable, and we adjusted our data for age, gender, smoking and alcohol consumption. We observed that blood type A was associated with a higher incidence of cutaneous malignant melanoma (OR = 1.575; 95% CI = 1.208–2.053).

At present, how ABO blood types trigger carcinogenesis is unclear. However, an explanation may be that the alleles for the ABO gene located on chromosome 9q34 encode three glycosyltransferases to form the antigenic structures of the ABO blood groups. Blood group antigens are present on key receptors, which differ according to the cancer type, and control cell proliferation, resistance to apoptosis and adhesion, such as receptors for integrins, cadherins, epidermal growth factor and CD44 [11]. Second, blood group antigens could influence two markers of inflammation–soluble intercellular adhesion molecule-1 (ICAM-1) and tumor necrosis factor-α (TNF-α) in plasma [20], [21]. ICAM-1 contributes to inflammatory responses within the blood vessel wall by augmenting atherosclerotic plaque formation and increasing endothelial cell activation. TNF-α can stimulate cell proliferation and differentiation under certain conditions. Some reports indicate that ICAM-1 and TNF-αare related to melanoma [22], [23]. Finally, a possible disequilibrium between the ABO gene and another gene may influence cancer risk.

To date, ours is the first study to investigate the relationship between ABO blood types and metastasis in patients with cutaneous malignant melanoma, although some studies have been undertaken analysis of such a relationship in tumors [24]–[26]. In one study of ABO blood groups and distant metastases in patients with nasopharyngeal carcinoma, the rate of distant metastases was significantly higher in male patients with blood type A than those with other blood types [24]. Prior studies [25], [26] indicate that having the O blood group prevented the spread of tumors.

We found that blood group A contributed to metastases of cutaneous malignant melanoma. So, ABO blood group may play different roles in tumor development. ABO blood group antigens are also expressed on cancer cells and can be modified by hypermethylation of the ABO gene promoter, a feature of malignant tumors. Such changes in the ABO blood antigens on cancer cells may be related to tumor invasiveness or metastatic potential.

In conclusion, we found that blood type A was associated with a higher incidence and metastasis of cutaneous malignant melanoma but future studies are needed to examine the mechanisms linking the cutaneous malignant melanoma risk to ABO blood types.

## References

[1] Lee C, Collichio F, Ollila D, Moschos S (2013) Historical review of melanoma treatment and outcomes. Clin Dermatol 31: 141–147. 23438377 10.1016/j.clindermatol.2012.08.015

[2] Moan J, Baturaite Z, Porojnicu AC, Dahlback A, Juzeniene A (2012) UVA, UVB and incidence of cutaneous malignant melanoma in Norway and Sweden. Photochem Photobiol Sci 11: 191–198. 21986949 10.1039/c1pp05215b

[3] Naldi L, Altieri A, Imberti GL, Gallus S, Bosetti C, et al (2005) Sun Exposure, Phenotypic Characteristics, and Cutaneous Malignant Melanoma. An Analysis According to Different Clinico-Pathological Variants and Anatomic Locations (Italian). Cancer Causes and Control 16: 893–899. 16132799 10.1007/s10552-005-2300-4

[4] Han J, Colditz GA, Hunter DJ (2006) Risk factors for skin cancers: a nested case-control study within the Nurses’ Health Study. Int J Epidemiol 35: 1514–1521. 16943234 10.1093/ije/dyl197

[5] Li Q, Yu CH, Yu JH, Liu L, Xie SS, et al (2012) ABO blood group and the risk of hepatocellular carcinoma: a case-control study in patients with chronic hepatitis B. PLOS ONE. 7: e29928. 10.1371/journal.pone.0029928 PMC3250489 22235351

[6] Song HR, Shin MH, Kim HN, Piao JM, Choi JS, et al (2013) Sex-specific differences in the association between ABO genotype and gastric cancer risk in a Korean population. Gastric Cancer 16: 254–260. 22865192 10.1007/s10120-012-0176-z

[7] Rizzato C, Campa D, Pezzilli R, Soucek P, Greenhalf W, et al (2013) ABO blood groups and pancreatic cancer risk and survival: results from the PANcreatic Disease ReseArch (PANDoRA) consortium. Oncol Rep 29: 1637–1644. 23403949 10.3892/or.2013.2285

[8] Poole EM, Gates MA, High BA, Chanock SJ, Cramer DW, et al (2012) ABO blood group and risk of epithelial ovarian cancer within the Ovarian Cancer Association Consortium. Cancer Causes Control 23: 1805–1810. 22961099 10.1007/s10552-012-0059-y PMC3474344

[9] Wang Z, Liu L, Ji J, Zhang J, Yan M, et al (2012) ABO Blood Group System and Gastric Cancer: A Case-Control Study and Meta-Analysis. Int J Mol Sci 13: 13308–13321. 23202954 10.3390/ijms131013308 PMC3497328

[10] Nakao M, Matsuo K, Ito H, Shitara K, Hosono S, et al (2011) ABO genotype and the risk of gastric cancer, atrophic gastritis, and Helicobacter pylori infection. Cancer Epidemiol Biomarkers Prev 20: 1665–1672. 21680535 10.1158/1055-9965.EPI-11-0213

[11] Ben Q, Wang K, Yuan Y, Li Z (2011) Pancreatic cancer incidence and outcome in relation to ABO blood groups among Han Chinese patients: a case-control study. Int J Cancer 128: 1179–1186. 20473916 10.1002/ijc.25426

[12] Curtin JA, Busam K, Pinkel D, Bastian BC (2006) Somatic activation of KIT in distinct subtypes of melanoma. J Clin Oncol 24: 4340–4346. 16908931 10.1200/JCO.2006.06.2984

[13] Curtin JA, Fridlyand J, Kageshita T, Patel HN, Busam KJ, et al (2005) Distinct sets of genetic alterations in melanoma. N Engl J Med 353: 2135–2147. 16291983 10.1056/NEJMoa050092

[14] Whiteman DC, Pavan WJ, Bastian BC (2011) The melanomas: a synthesis of epidemiological, clinical, histopathological, genetic, and biological aspects, supporting distinct subtypes, causal pathways, and cells of origin. Pigment Cell Melanoma Res 24: 879–897. 21707960 10.1111/j.1755-148X.2011.00880.x PMC3395885

[15] Aird I, Bentall HH, Roberts JA (1953) A relationship between cancer of stomach and the ABO blood groups. Br Med J 1: 799–801. 13032504 10.1136/bmj.1.4814.799 PMC2015995

[16] Greer JB, Yazer MH, Raval JS, Barmada MM, Brand RE, et al (2010) Significant association between ABO blood group and pancreatic cancer. World J Gastroenterol 16: 5588–5591. 21105191 10.3748/wjg.v16.i44.5588 PMC2992676

[17] Gates MA, Wolpin BM, Cramer DW, Hankinson SE, Tworoger SS (2011) ABO blood group and incidence of epithelial ovarian cancer. Int J Cancer 128: 482–486. 20309936 10.1002/ijc.25339 PMC2946962

[18] Xie J, Qureshi AA, Li Y, Han J (2010) ABO blood group and incidence of skin cancer. PLOS ONE 5: e11972. 20694147 10.1371/journal.pone.0011972 PMC2915921

[19] de Giorgi V, Grazzini M, Gori A, Alfaioli B, Rossari S, et al (2011) ABO blood group and risk of cutaneous malignant melanoma. Eur J Cancer Prev 20: 121–122. 21332097 10.1097/cej.0b013e3283429e1d

[20] Pare G, Chasman DI, Kellogg M, Zee RY, Rifai N, et al (2008) Novel association of ABO histo-blood group antigen with soluble ICAM-1: results of a genome - wide association study of 6,578 women. PLoS Genet 4: e1000118. 18604267 10.1371/journal.pgen.1000118 PMC2432033

[21] Melzer D, Perry JR, Hernandez D, Corsi AM, Stevens K, et al (2008) A genome-wide association study identifies protein quantitative trait loci (pQTLs). PLoS Genet 4: e1000072. 18464913 10.1371/journal.pgen.1000072 PMC2362067

[22] Yamada M, Yanaba K, Takehara K, Sato S (2005) Clinical significance of serum levels of soluble intercellular adhesion molecule-1 and soluble L-selectin in malignant melanoma. Arch Dermatol Res 297: 256–260. 16222535 10.1007/s00403-005-0605-5

[23] de Lima VC, de Carvalho AF, Morato-Marques M, Hashimoto VL, Spilborghs GM, et al (2013) TNF-alpha and melphalan modulate a specific group of early expressed genes in a murine melanoma model. Cytokine 62: 217–225. 23534980 10.1016/j.cyto.2013.02.022

[24] Sheng L, Sun X, Zhang L, Su D (2013) ABO blood group and nasopharyngeal carcinoma risk in a population of Southeast China. Int J Cancer 133: 893–897. 23389798 10.1002/ijc.28087

[25] Beckman L, Angqvist KA (1987) On the mechanism behind the association between ABO blood groups and gastric carcinoma. Hum Hered 37: 140–143. 3583294 10.1159/000153691

[26] Jaff MS (2010) Higher frequency of secretor phenotype in O blood group - its benefits in prevention and/or treatment of some diseases. Int J Nanomedicine 5: 901–905. 21116330 10.2147/IJN.S13980 PMC2990383

